# Impact of Placental Grading on Pregnancy Outcomes: A Retrospective Cohort Study

**DOI:** 10.3390/healthcare13060601

**Published:** 2025-03-10

**Authors:** Antonios Siargkas, Ioannis Tsakiridis, Georgios Michos, Anastasios Liberis, Sofoklis Stavros, Menelaos Kyriakakis, Ekaterini Domali, Apostolos Mamopoulos, Themistoklis Dagklis

**Affiliations:** 1 Third Department of Obstetrics and Gynecology, Faculty of Health Sciences, School of Medicine, Aristotle University of Thessaloniki, 54124 Thessaloniki, Greece; antonis.siargkas@gmail.com (A.S.); themistoklis.dagklis@gmail.com (T.D.); 2 Third Department of Obstetrics and Gynecology, University Hospital “ATTIKON”, Medical School of the National, Kapodistrian University of Athens, 11527 Athens, Greece; 3 First Department of Obstetrics and Gynecology, National and Kapodistrian University of Athens, 11527 Athens, Greece

**Keywords:** placental calcification, placental grading, Grannum grading, preeclampsia, SGA, FGR, stillbirth

## Abstract

Background: Placental grading remains underutilized in clinical practice despite its potential prognostic value. This study aimed to elucidate the relationship between premature placental calcification (PPC) and relevant perinatal outcomes in a large cohort. Methods: We conducted a retrospective cohort study involving 3088 singleton pregnancies that underwent routine third-trimester ultrasound examinations (30^+0^ to 35^+6^ gestational weeks) at the Third Department of Obstetrics and Gynecology, School of Medicine, Faculty of Health Sciences, Aristotle University of Thessaloniki, Greece, between January 2018 and December 2023. Placental calcification was graded using the Grannum system, categorizing placentas into Grades 0–1 (control), Grade 2, and Grade 3. Primary outcomes assessed were small for gestational age neonates (SGA) and preeclampsia. Secondary outcomes included gestational hypertension, fetal growth restriction (FGR), stillbirth, gestational age at birth, and birthweight centile. Multiple logistic regression was employed to adjust for confounders, i.e., maternal age, BMI, smoking, conception via assisted reproductive technology, and uterine artery pulsatility index. Results: In total, 544 pregnancies (17.6%) had Grade 2 placentas, and 41 pregnancies (1.3%) had Grade 3 placentas. Compared to the control group, Grade 2 placentas were associated with increased odds of SGA (adjusted odds ratio [aOR] 1.80; 95% confidence intervals [CI]: 1.43–2.25) and FGR (aOR 1.81; 95% CI: 1.35–2.42). Grade 3 placentas showed even higher odds of SGA (aOR 3.09; 95% CI: 1.55–6.17) and FGR (aOR 3.26; 95% CI: 1.53–6.95). No significant associations were found between placental grading and preeclampsia or stillbirth. Additionally, PPC was linked to lower birthweight percentiles and earlier gestational age at birth. Conclusions: Premature placental calcification (before 36^+0^ weeks), particularly Grade 3, is significantly associated with adverse perinatal outcomes such as SGA and FGR. Incorporating placental grading into routine prenatal care may enhance risk stratification and guide clinical decision making beyond traditional assessment methods.

## 1. Introduction

As gestation progresses, normal aging leads to detectable changes in the placental appearance on ultrasound imaging; Winsberg was among the first to link ultrasonically detectable placental changes with fetal maturity [1]. Later, a grading system was developed to assess placental maturation and its subsequent increased calcification, correlating a Grade 3 placenta with fetal pulmonary maturity at birth [2]. Furthermore, premature placental calcification (PPC), defined as increased placental calcification (Grannum Grade 3 or Grade 2 and 3 combined) detected before the 36th week of gestation, has been associated with fetal growth restriction (FGR) [3,4,5,6,7,8], preeclampsia [3,8], low Apgar score (<7 at 5 min) [6,9], stillbirth [10,11] and perinatal death [9]. While many of these studies reported significant associations with specific outcomes, they failed to replicate the significant findings of other studies, leading to considerable controversies [7,8,12,13,14]. The only relevant meta-analysis did not specifically examine PPC but considered Grade 3 placentas in general, without specific exclusion criteria, making its findings difficult to generalize [15]. It found that pregnancies with a placental Grade of 3 were at an increased risk for perinatal death, fetal compromise, meconium liquor, and low gestational weight [15].

In recent years, research on placental grading has significantly declined, despite its potential clinical importance. Possible reasons include limitations such as interobserver variability, ambiguity in the Grannum grading system, and differences in ultrasonographic device capabilities, which may deter researchers. However, the published data suggest that PPC may gradually narrow placental vessels, potentially affecting perinatal outcomes through mechanisms such as basement membrane mineralization, acute atherosclerosis, and focal vascular calcification [16,17,18].

Building on this foundation, our study aimed to be the first large-scale cohort to examine the association between PPC and various perinatal outcomes. By adjusting for key confounders, including the mean Uterine Artery Pulsatility Index (UtA PI), we sought to provide precise effect estimates and assess its individual contribution to adverse outcomes.

## 2. Materials and Methods

### 2.1. Study Design and Population

This retrospective cohort study included women who underwent routine ultrasound examinations between 30^+0^ and 35^+6^ weeks of gestation at the Third Department of Obstetrics and Gynecology, School of Medicine, Faculty of Health Sciences, Aristotle University of Thessaloniki, Greece, from 2 January 2018 to 30 December 2023. According to the national guidelines, all women are offered 3 scans, at 11^+0^–13^+6^ weeks, at 20^+0^–23^+6^ weeks, and at 30^+0^–36^+6^ weeks, the latter for growth, placenta, and anatomy check. The grading of the placenta was routinely recorded but did not guide clinical practice. Eligible participants were women with singleton pregnancies carrying a live fetus within the specified gestational age range, without known genetic anomalies or major fetal defects diagnosed either before or after birth. We excluded pregnancies where genetic anomalies or major fetal defects were identified prenatally or postnatally, as well as pregnancies lost to follow-up. All sonographers involved were certified by the Fetal Medicine Foundation (FMF), UK. All the measurements were performed with a Voluson E8, (GE Healthcare, Pfaffing, Austria), or a WS80 Elite (Samsung, South Korea) machine.

Women consented to the anonymity of their data and their potential use for future research, with no incentives provided. Following the policy for observational studies that do not involve any interventions or modifications to routine patient care, no institutional board review was required for this study [19].

At the initial consultation, we collected comprehensive maternal information, including height, weight, and sociodemographic details such as age, parity, smoking status, and medical and obstetrical history. During the third-trimester ultrasound, the estimated fetal weight was calculated using the Hadlock formula, incorporating the head circumference, abdominal circumference, and femur length. Doppler assessments were performed to evaluate the fetal blood flow, measuring the uterine, umbilical, and middle cerebral artery indices, as well as the ductus venosus pulsatility index. Additionally, detailed ultrasonographic evaluations were conducted to identify placental and umbilical cord abnormalities, including an assessment of placental calcification using Grannum grading [2].

Our primary outcomes under investigation were the relationship of PPC, defined as Grannum grading 2 or 3 between 30^+0^ and 35^+6^ weeks of gestation, with small for gestational age neonates at birth (SGA) defined as <10th percentile and preeclampsia [20]. These outcomes were selected as they are among the most common and characteristic indicators of placental dysfunction. Secondary outcomes were gestational hypertension, fetal growth restriction (FGR), stillbirth, gestational age at birth (weeks), and birthweight centile. Fetal growth restriction was defined according to the Delphi consensus by Gordijn et al. [21].

Singleton pregnancies with placental Grades 0 or 1 served as the control group. Two distinct study groups were designated: one group consisted of pregnancies with a Grade 3 placenta and the other group with a Grade 2 placenta.

This study was designed and reported in accordance with the Strengthening the Reporting of Observational Studies in Epidemiology (STROBE) guidelines [22].

### 2.2. Statistical Analysis

Continuous variables were assessed for normality through visual inspection using histograms and Q-Q plots. Variables that approximated a normal distribution are presented as means and their 95% CIs and were compared among the three groups using one-way Analysis of Variance (ANOVA). Non-normally distributed continuous variables are expressed as median and their 95% CIs and were compared using the Kruskal–Wallis test. Categorical variables are presented as percentages and their CIs, with comparisons made using the Chi-square test.

We applied multiple logistic regression to the original cohort to calculate the adjusted odds ratio (aOR) with its 95% confidence interval (CI), exploring the potential association of PPC with various perinatal outcomes. The primary confounding factors included in our models were maternal age, parity, use of assisted reproductive technology (ART), smoking, body mass index (BMI), and mean UtA PI percentile. Additional confounders were incorporated when the sample size allowed, adhering to the rule of at least 10 study cases per independent variable, and were selected based on clinical relevance.

Statistical analyses were conducted and reported following the Transparent Reporting of a Multivariable Prediction Model for Individual Prognosis or Diagnosis (TRIPOD) guidelines [23]. A *p*-value of less than 0.05 was considered statistically significant. All analyses were performed using R software version 2.15.1 [24]. The stats package in R was utilized for multiple logistic regression [24].

## 3. Results

**Figure 1:**
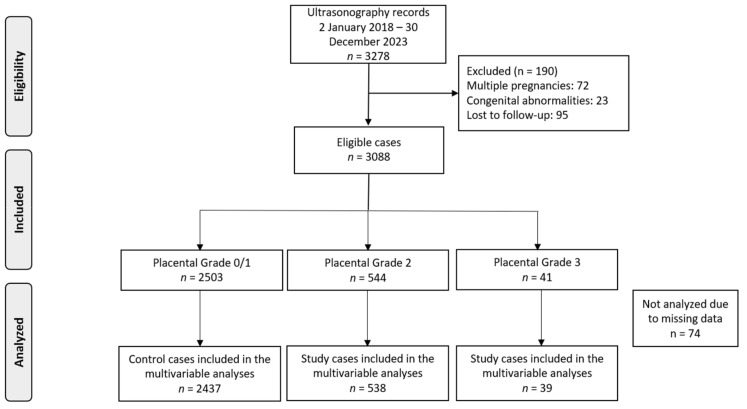
Flowchart of patient inclusion in the study.

In total, 3278 pregnancies attended our clinics during the study period. Following the exclusion of certain cases due to factors like multiple pregnancies (*n* = 72), congenital abnormalities (*n* = 23), and lost to follow-up (*n* = 95), our analysis was refined to focus on 3088 singleton pregnancies (Figure 1). The dataset had minimal missing data (*n* = 74), which we believe occurred at random, likely due to the retrospective nature of the study.

There were 41 pregnancies with Grade 3 placenta (1.3%) and 544 pregnancies with a Grade 2 placenta (17.6%) at the third-trimester ultrasound examination from 30^+0^ to 35^+6^ weeks of gestation (Table 1). Compared to the control group, women with PPC were more likely to be younger, nulliparous, smokers, and to have pregnancies conceived via ART.

### Investigated Outcomes

Regarding the primary outcomes, when compared to placentas with Grade 0 or 1, pregnancies with Grade 2 placentas were associated with almost double odds of SGA neonates (aOR 1.80; 95% CI: 1.43, 2.25), and Grade 3 placentas increased the odds approximately three-fold (aOR 3.09; 95% CI: 1.55, 6.17) (Table 2). Regarding preeclampsia, both Grade 2 (aOR 1.86; 95% CI: 0.83, 4.15) and Grade 3 (aOR 2.40; 95% CI: 0.55, 10.46) placentas were not significantly associated, compared to the control group (Table 3).

Regarding the rest of the investigated outcomes, gestational hypertension was not significantly associated with Grade 2 placentas (aOR 1.19; 95% CI: 0.68, 2.10), nor with Grade 3 placentas (aOR 2.03; 95% CI: 0.64, 6.49) (Supplementary Table S1). For stillbirth, both Grade 2 (aOR 1.25; 95% CI: 0.14, 11.47) and Grade 3 (aOR 13.34; 95% CI: 0.88, 201.14) placentas showed no statistically significant association (Supplementary Table S2). FGR was significantly associated with Grade 2 (aOR 1.81; 95% CI: 1.35, 2.42) and Grade 3 placentas (aOR 3.26; 95% CI: 1.53, 6.95) (Supplementary Table S3). Grade 2 placentas were significantly associated with a reduction in birthweight percentiles, decreasing the mean by 10.76 percentiles (95% CI: −13.66 to −7.87), and Grade 3 placentas were also associated with a mean decrease of 11.60 percentiles (95% CI: −21.26 to −1.94) (Supplementary Table S4). Regarding gestational age at birth, Grade 2 placentas were linked to an average decrease of 2 days (95% CI: −0.41, −0.15), while Grade 3 placentas were associated with an average reduction of 9 days (95% CI: −1.78, −0.89) (Supplementary Table S5). The cumulative results are presented in Table 4.

## 4. Discussion

### 4.1. Main Findings

This study’s main findings underscore the clinical significance of placental calcification before term. Specifically, we found that from 30^+0^ to 35^+6^ weeks (i) the prevalence of Grade 2 and Grade 3 placentas was 17.6% and 1.3%, respectively; (ii) PPC was strongly associated with several adverse perinatal outcomes, including SGA neonates, FGR, lower birthweight centile; and earlier gestational age at birth (iii); Grade 3 placentas showed the highest risk for these adverse outcomes; and (iv) no significant association between placental grading and preeclampsia or stillbirth was identified.

### 4.2. Interpretation of the Findings

Placental calcification has long been considered a potential indicator of compromised placental function, particularly in the third trimester. Despite its clinical relevance, placental grading has not been universally adopted in obstetric practice, with UtA PI currently being the standard for evaluating placental dysfunction during the third trimester. Our findings, however, suggest that placental grading provides independent prognostic value, beyond UtA PI, for key outcomes such as SGA and FGR.

In agreement with previous studies, our results showed that Grade 3 placental calcification was associated with a more than three-fold increase in the odds of both SGA and FGR. Furthermore, neonates with Grade 2 and Grade 3 placentas had birthweights that were significantly lower, with an average reduction of 10.8 and 11.6 percentiles, respectively. These findings are consistent with other studies that have reported a two- to three-fold increase in the risk of FGR and SGA in pregnancies with Grade 3 placental calcification (RR 1.94, 95% CI: 1.26, 3.00; RR 2.4, 95% CI: 1.33, 15.61, respectively) [3,8,11,13,14]. One possible explanation for these associations is that placental calcification impairs placental function, potentially reducing the blood flow and nutrient supply to the fetus. Pathological studies suggest that calcium and fibrin deposits can obstruct placental blood vessels, leading to impaired circulation [25]. This is supported by research showing significant mineralization of the basement membrane in placentas from fetal Bartter syndrome, where calcification and signs of atherosclerosis in placental vessels have been observed [16,17]. Furthermore, calcifications in the basal plate of the placenta have been linked to maternal floor infarction [26], a condition associated with FGR and mid-trimester pregnancy loss [27]. Other researchers have observed calcifications and blood clots that block the chorionic and umbilical vessels, reinforcing the idea that these blockages play a key role in PPC-related growth restriction [28,29]. These findings suggest that PPC, rather than being a normal part of pregnancy, may represent a pathological process contributing to adverse outcomes. Moreover, on a molecular level, placental aging is strongly linked to FGR and SGA outcomes through mechanisms such as telomere attrition, reduced telomerase activity, and mitochondrial dysfunction, which drive cellular senescence and oxidative stress [30]. These processes impair placental function by disrupting nutrient transport pathways (e.g., reduced mTORC1/mTORC2 activity) and promoting apoptosis via upregulated p53 expression, ultimately contributing to suboptimal fetal growth [30]. Regarding possible biomarkers of placental aging and dysfunction, one angiogenic and anti-angiogenic marker is the reduced placental growth factor (PlGF) and elevated sFlt-1/PlGF ratio, both strongly associated with placental insufficiency and SGA risk [31]. Additionally, Pregnancy-Associated Plasma Protein-A, a placental glycoprotein, is a marker of placental development, with low levels indicating impaired placental function [31]. Oxidative stress and hypoxia, hallmarks of placental aging, are reflected in the Hypoxia-Inducible Factor 1-alpha marker, which correlates with telomere attrition, reduced nutrient transport, and accelerated cellular senescence [32].

Of note, our study did not confirm previous reports linking placental calcification with preeclampsia or gestational hypertension. Several studies have found significant associations between Grade 3 placental calcification and preeclampsia (RR 1.7, 4.7, and 12.3) [3,8,14]; yet, in our analysis, these associations lost statistical significance after adjusting for UtA PI and other confounders. This highlights the dominant role of UtA PI in predicting hypertensive disorders in the third trimester, suggesting that placental calcification may not be an independent risk factor for preeclampsia.

Regarding stillbirth, our analysis showed that Grades 2 or 3 had an increased effect measure, but this association did not reach statistical significance. A large prospective study of 15,122 cases reported a statistically significant (RR 7.62) risk for stillbirth associated with Grade 3 placenta [10]. Another study found a significant decrease in the risk of perinatal death when the placental grading was known to the clinician [9]. This disparity may be partly explained by our adjustment for confounders, as well as the implementation of systematic prenatal surveillance and timely interventions, such as prompt induction of labor, which may help in mitigating the risk of stillbirth despite poor placental grading. This is supported by previous studies that indicated that the use of ultrasound-based placental assessments and Doppler velocimetry can guide obstetric decisions, potentially improving perinatal outcomes by facilitating earlier intervention in cases of FGR and SGA [33,34]. Furthermore, the limited number of stillbirth cases in our sample may have reduced the statistical power to detect a significant effect for this outcome.

Our analysis also revealed that PPC is associated with earlier delivery. Grade 2 placentas were linked to a modest decrease in the mean gestational age at birth (2 days), while Grade 3 placentas were associated with a more substantial reduction of 9 days. While these results are statistically significant, it is important to consider the potential for observer and treatment bias regarding the timing of delivery. Physicians may be more likely to induce labor or deliver earlier in cases with ischemic placental disease, especially if other signs of fetal distress are present.

### 4.3. Possible Clinical Implications

This study highlights the clinical importance of PPC. While UtA PI remains a critical tool for assessing fetal risk, placental grading—especially the identification of Grade 3 placentas—offers additional prognostic value. Incorporating placental grading into routine prenatal care could help clinicians better identify high-risk pregnancies and guide decision making. Pregnancies with PPC may warrant closer surveillance for early detection of placental insufficiency, but this needs further investigation.

### 4.4. Strengths and Limitations

A key strength of this study is its large cohort, which allowed for robust statistical analysis and adjustment for multiple confounders, including maternal age, BMI, smoking, conception via ART, and UtA PI. This enhances the reliability of our findings. Additionally, all sonographers were FMF-certified, ensuring high-quality ultrasound assessments. The study followed rigorous reporting guidelines (STROBE and TRIPOD), ensuring transparency and methodological rigor.

However, the retrospective design of the study limits causality, and the single-center data may reduce generalizability. The small number of stillbirths in our sample may have limited the power to detect significant associations for this outcome. Although we adjusted for several confounders, residual confounding remains a concern. Furthermore, potential inter-observer variability on the placental grading may have affected our results and thus may be associated with bias; however, the sample size is large enough, and all the sonographers were FMF certified, which increases the generalizability of the findings.

## 5. Conclusions

This study highlights the clinical importance of placental calcification before term. While UtA PI remains a critical tool for assessing fetal risk, placental grading—especially the identification of Grade 3 placentas—offers additional prognostic value. Incorporating placental grading into routine prenatal care could help clinicians better identify high-risk pregnancies and guide decision making, particularly in cases with suspected ischemic placental disease. Further research is needed to explore how placental grading can be integrated with other monitoring tools to enhance outcomes.

## Figures and Tables

**Table 1:** Characteristics of the population and our investigated groups.

Variable	All (*n* = 3088)	Grade 0 or 1 (*n* = 2503)	Grade 2 (*n* = 544)	Grade 3 (*n* = 41)	*p*-Value
Maternal Age *	32.48 (32.29;32.66)	32.67 (32.47;32.88)	31.73 (31.26;32.19)	30.30 (28.69;31.92)	<0.001
BMI ^†^	22.77 [22.66;22.96]	22.95 [22.72;23.11]	22.44 [22.10;22.66]	22.55 [21.16;24.92]	0.026
Multiparity ^‡^	36.69% [34.99%;38.42%]	37.48% [35.57%;39.41%]	34.01% [30.03%;38.16%]	24.39% [12.36%;40.30%]	0.081
ART ^‡^	6.31% [5.48%;7.23%]	6.55% [5.61%;7.59%]	4.96% [3.30%;7.14%]	9.76% [2.72%;23.13%]	0.210
Smoking ^‡^	11.27% [10.18%;12.44%]	8.43% [7.37%;9.59%]	23.53% [20.02%;27.32%]	21.95% [10.56%;37.61%]	<0.001
Preexisting diabetes mellitus ^‡^	0.32% [0.16%;0.59%]	0.32% [0.14%;0.63%]	0.37% [0.04%;1.32%]	0.00% [0.00%;8.60%]	0.734
Chronic hypertension ^‡^	0.52% [0.30%;0.84%]	0.48% [0.25%;0.84%]	0.74% [0.20%;1.87%]	0.00% [0.00%;8.60%]	0.603
Stillbirth ^‡^	0.29% [0.13%;0.55%]	0.28% [0.11%;0.58%]	0.18% [<0.01%;1.02%]	2.44% [0.06%;12.86%]	0.124
Preeclampsia ^‡^	1.13% [0.79%;1.57%]	0.88% [0.55%;1.33%]	1.84% [0.88%;3.35%]	7.32% [1.54%;19.92%]	0.003
Mean UtA percentile ^†^	59.50 [57.87;61.06] NA = 74	58.42 [56.74;60.15] NA = 66	65.35 [59.66;68.82] NA = 6	88.26 [55.00;97.74] NA = 2	<0.001
Gestational hypertension ^‡^	2.95% [2.38%;3.61%]	2.72% [2.12%;3.43%]	3.49% [2.12%;5.40%]	9.76% [2.72%;23.13%]	0.031
SGA ^‡^	20.05% [18.65%;21.50%]	17.38% [15.91%;18.92%]	30.33% [26.49%;34.39%]	46.34% [30.66%;62.58%]	<0.001
FGR ^‡^	9.72% [8.69%;10.81%]	7.95% [6.92%;9.08%]	16.18% [13.18%;19.54%]	31.71% [18.08%;48.09%]	<0.001
Gestational diabetes ^‡^ mellitus	17.94% [16.60%;19.34%]	17.98% [16.49%;19.54%]	17.46% [14.36%;20.92%]	21.95% [10.56%;37.61%]	0.765
Gestational age at birth (weeks) ^†^	38.86 [38.86;39.00]	39.00 [39.00;39.00]	38.64 [38.43;38.86]	37.29 [36.71;38.29]	<0.001
BW percentile ^†^	41.62 [39.63;43.74]	45.89 [43.42;49.20]	24.25 [20.20;29.48]	10.67 [3.07;44.84]	<0.001

Abbreviations: ART, assisted reproductive technology; BW, birth weight; SGA, small for gestational age; FGR, fetal growth restriction; UtA, uterine artery pulsatility index; NA, not available. *, normal continuous variable expressed as mean and 95% confidence intervals, compared with one-way Analysis of Variance; ^†^, non-normal continuous variable expressed as median and 95% confidence intervals, compared with Kruskal–Wallis; ^‡^, categorical variable expressed as percentage and 95% confidence intervals, compared with Chi-square test.

**Table 2:** Multivariable logistic regression investigating the relationship between placental grading and odds of small for gestational age neonates.

Variable	aOR	95% CI	*p*-Value
Placental grading (Grade 0 and 1 as reference)			
Grade 2	1.795	1.433, 2.249	0.000
Grade 3	3.093	1.552, 6.165	0.001
Maternal age (Years)	1.000	0.981, 1.019	1.000
BMI (kg/m^2^)	0.939	0.920, 0.960	0.000
Multiparity	0.600	0.463, 0.777	0.000
ART	0.910	0.607, 1.364	0.647
Smoking	1.432	1.083, 1.895	0.012
Mean UtA PI percentile	1.530	1.406, 1.666	0.000
History of SGA	5.125	2.008, 13.081	0.001

Abbreviations: ART, assisted reproductive technology; SGA, small for gestational age neonates; UtA PI, uterine artery pulsatility index.

**Table 3:** Multivariable logistic regression investigating the relationship between placental grading and odds of preeclampsia.

Variable	aOR	95% CI	*p*-Value
Placental grading (Grade 0 and 1 as reference)			
Grade 2	1.857	0.832, 4.147	0.131
Grade 3	2.400	0.551, 10.462	0.244
Maternal age (Years)	1.077	0.997, 1.163	0.061
BMI (kg/m^2^)	1.066	1.007, 1.128	0.027
Multiparity	0.788	0.365, 1.704	0.545
ART	2.804	1.000, 7.865	0.050
Smoking	0.238	0.032, 1.790	0.163
Mean UtA PI percentile	2.651	2.051, 3.426	0.000
History of preeclampsia	5.156	0.937, 28.368	0.059

Abbreviations: ART, assisted reproductive technology; UtA PI, uterine artery pulsatility index.

**Table 4:** Cumulative results of the multivariable regressions on the association of premature placental calcification and the investigated outcomes.

Outcome	Grade 2 aOR (95% CI)	Grade 2 *p*-Value	Grade 3 aOR (95% CI)	Grade 3 *p*-Value
SGA <10th Percentile	1.80 (1.43, 2.25)	<0.001	3.09 (1.55, 6.17)	0.001
Preeclampsia	1.86 (0.83, 4.15)	0.131	2.40 (0.55, 10.46)	0.244
Gestational hypertension	1.19 (0.68, 2.10)	0.544	2.03 (0.64, 6.49)	0.231
Stillbirth	1.25 (0.14, 11.47)	0.843	13.34 (0.88, 201.14)	0.061
FGR	1.81 (1.35, 2.42)	<0.001	3.26 (1.53, 6.95)	0.002
Birthweight percentile	−10.76 (−13.66, −7.87)	<0.001	−11.60 (−21.26, −1.94)	0.019
Gestational age at birth (weeks)	−0.28 (−0.41, −0.15)	<0.001	−1.34 (−1.78, −0.89)	<0.001

Abbreviations: SGA, small for gestational age; FGR, fetal growth restriction. Multivariable logistic or linear regression adjusting at least for maternal age, BMI, multiparity, assisted reproductive technology, smoking and mean uterine artery pulsatility index percentile.

## Data Availability

Data are available upon reasonable request.

## Supplementary Materials

The following supporting information can be downloaded at https://www.mdpi.com/article/10.3390/healthcare13060601/s1, Supplementary Table S1: Multivariable logistic regression investigating the relationship between placental grading and odds of gestational hypertension. Supplementary Table S2: Multivariable logistic regression investigating the relationship between placental grading and odds of stillbirth. Supplementary Table S3: Multivariable logistic regression investigating the relationship between placental grading and odds of fetal growth restriction. Supplementary Table S4: Multivariable logistic regression investigating the relationship between placental grading and birthweight percentile. Supplementary Table S5: Multivariable logistic regression investigating the relationship between placental grading and gestational age at birth measured in weeks.
