# Supplementary material for: Impact of Placental Grading on Pregnancy Outcomes: A Retrospective Cohort Study
Source: Healthcare (Basel). 2025 Mar 10;13(6):601. doi: 10.3390/healthcare13060601 (PMC11942256; doi:10.3390/healthcare13060601)
Supplement: Supplementary file 1 [file healthcare-13-00601-s001.zip › healthcare-3478129-supplementary.pdf]

Supplementary Table 1. Multivariable logistic regression investigating the relationship between placental grading and odds of gestational hypertension.

Variable	aOR	95% CI	p-value
Placental grading (Grade 0 & 1 as reference)			
Grade 2	1.191	0.676, 2.098	0.544
Grade 3	2.034	0.637, 6.492	0.231
Maternal age (Years)	0.990	0.945, 1.037	0.676
BMI (kg/m^2)	1.100	1.063, 1.139	0.000
Multiparity	0.446	0.256, 0.779	0.005
ART	2.255	1.067, 4.766	0.033
Smoking	0.666	0.296, 1.495	0.325
Mean UtA PI percentile	1.676	1.408, 1.994	0.000
History of preeclampsia	6.098	1.563, 23.794	0.009

Abbreviations: ART, assisted reproductive technology; UtA PI, uterine artery pulsatility index

Supplementary Table 2. Multivariable logistic regression investigating the relationship between placental grading and odds of stillbirth.

Variable	aOR	95% CI	p-value
Placental grading (Grade 0 & 1 as reference)			
Grade 2	1.251	0.137, 11.465	0.843
Grade 3	13.337	0.884, 201.135	0.061
Maternal age (Years)	1.132	0.935, 1.369	0.204
BMI (kg/m^2)	1.052	0.924, 1.199	0.443
Multiparity	1.108	0.210, 5.844	0.904
ART	0.000	0.000, Inf	0.996
Smoking	0.000	0.000, Inf	0.995
Mean UtA PI percentile	1.495	0.807, 2.769	0.201

Abbreviations: ART, assisted reproductive technology; UtA PI, uterine artery pulsatility index

Supplementary Table 3. Multivariable logistic regression investigating the relationship between placental grading and odds of fetal growth restriction.

Variable	aOR	95% CI	p-value
Placental grading (Grade 0 & 1 as reference)			
Grade 2	1.808	1.350, 2.421	0.000
Grade 3	3.255	1.525, 6.950	0.002

Maternal age (Years)	0.995	0.969, 1.021	0.680
BMI (kg/m²)	0.944	0.918, 0.972	0.000
Multiparity	0.777	0.585, 1.030	0.080
ART	1.292	0.774, 2.158	0.327
Smoking	1.581	1.105, 2.263	0.012
Mean UtA PI percentile	1.758	1.579, 1.956	0.000
History of SGA	7.269	2.788, 18.954	0.000

Abbreviations: ART, assisted reproductive technology; SGA, small for gestational age neonates; UtA PI, uterine artery pulsatility index

Supplementary Table 4. Multivariable logistic regression investigating the relationship between placental grading and birthweight percentile.

Variable	aOR	95% CI	p-value
Placental grading (Grade 0 & 1 as reference)			
Grade 2	-10.761	-13.656, -7.867	0.000
Grade 3	-11.598	-21.259, -1.937	0.019
Maternal age (Years)	-0.056	-0.279, 0.168	0.624
BMI (kg/m²)	1.043	0.825, 1.261	0.000
Multiparity	6.857	4.435, 9.279	0.000
ART	2.533	-2.158, 7.225	0.290
Smoking	-5.766	-9.261, -2.272	0.001
Mean UtA PI percentile	-6.092	-7.111, -5.073	0.000
History of SGA	-24.090	-37.202, -10.979	0.000

Abbreviations: ART, assisted reproductive technology; SGA, small for gestational age neonates; UtA PI, uterine artery pulsatility index

Supplementary Table 5. Multivariable logistic regression investigating the relationship between placental grading and gestational age at birth measured in weeks.

Variable	Estimate	95% CI	p-value
Placental grading (Grade 0 & 1 as reference)			
Grade 2	-0.278	-0.410, -0.145	<0.001
Grade 3	-1.335	-1.777, -0.892	<0.001
Maternal age (Years)	-0.006	-0.017, 0.004	0.225
BMI (kg/m²)	-0.013	-0.023, -0.003	0.011
Multiparity	0.087	-0.042, 0.216	0.184
ART	-0.473	-0.688, -0.259	<0.001
Smoking	-0.065	-0.225, 0.095	0.425
Mean UtA PI percentile	-0.241	-0.287, -0.194	<0.001

History of PTB	-0.687	-0.960, -0.414	<0.001

Abbreviations: ART, assisted reproductive technology; PTB, preterm birth; UtA PI, uterine artery pulsatility index
